# TGF-β signaling regulates *SPOP* expression and promotes prostate cancer cell stemness

**DOI:** 10.18632/aging.103085

**Published:** 2020-05-01

**Authors:** Chenchen Jiao, Tong Meng, Chenyu Zhou, Xinbo Wang, Ping Wang, Meiling Lu, Xiao Tan, Qing Wei, Xin Ge, Jiali Jin

**Affiliations:** 1 Tongji University Cancer Center, Shanghai Tenth People’s Hospital, Tongji University School of Medicine, Shanghai 200072, China; 2 Department of Pathology, Shanghai Tenth People's Hospital of Tongji University, Shanghai 200072, China; 3 Department of Clinical Medicine, Shanghai Tenth People's Hospital of Tongji University, Shanghai 200072, China; 4 School of Basic Medical Science, Ningxia Medical University, Yinchuan 75004, Ningxia, China; 5 Cancer and Aging Research Institute, School of Life Sciences, Shandong University of Technology, Zibo 255049, Shandong, China; 6 Department of Central Laboratory, School of Life Science and Technology, Shanghai Tenth People's Hospital of Tongji University, Tongji University, Shanghai 200072, China

**Keywords:** prostate cancer, TGF-β signaling, SMAD3, SPOP, stemness

## Abstract

SPOP, a substrate binding adaptor of E3 ubiquitin ligase Cullin3, is frequently mutated in human prostate cancer (PCa). However, whether and how SPOP is regulated at transcriptional level in PCa remain unclear. Here, we report that *SPOP* is down-regulated in PCa stem-like cells (CSCs) and tissues. Our study reveals that SPOP expression is repressed by TGF-β / SMAD signaling axis in PCa CSCs. *SPOP* promoter contains SMAD-binding elements (SBEs), which can interact with SMAD3. Moreover, TGF-β signaling inhibitor SB431542 promotes the SPOP expression and abrogates PCa stemness. Clinically, SPOP expression is downregulated in PCa patients, which is significantly related to a poor prognosis and lower survival rate. Thus, our findings uncover a mechanism of how SPOP expression is mediated in PCa CSCs via TGF-β/ SMAD3 signaling.

## INTRODUCTION

Cancer stem cells (CSCs) have been identified in PCa as well as a number of other solid tumors [1]. Accumulating evidence indicate that such CSCs account for PCa initiation, progression and resistance to chemotherapies [2]. Therefore, in-depth understanding about the regulatory mechanism unique to CSCs will be essential for getting to the root of cancer initiation / progression, and consequently, designing CSCs-specific therapeutics.

Transforming growth factor-beta (TGF-β) plays a crucial role in cell proliferation and differentiation [3]. TGF-β family members cooperate with membrane receptor serine-threonine protein kinase, leading to the activation of Smad transcription factors (TFs). Emerging evidence show that TGF-β has a complex and paradoxical role in cancer, acting as both a tumor suppressor and a factor that promotes cancer invasion and metastasis by suppressing immune responses [3–5]. TGF-β drives immune evasion in genetically reconstituted colon cancer metastasis by promoting T-cell exclusion and blocking the acquisition of the Th1-effector phenotype [6]. Thus, TGF-β signaling regulates tumorigenesis via different molecular mechanisms. Especially, TGF-β signaling plays an important role in regulating the function of cancer stem cells. As previous studies reported, TGF-β signaling is responsible for maintaining the tumorigenic properties of tumor-initiating cells in multiple tumors such as breast, melanoma, glioma and so on [7–9]. CSCs also take an important part in facilitating cancer metastases [10]. Recent studies reveal that inhibition of TGF-β signaling in PCa cells impedes the PCa progression and corresponding bone metastases [11–13], as well as the discovery that external stimulation with TGF-β converted CD44^-^ non-CSCs into the undifferentiated CD44^+^ CSCs in human colorectal cancer, leading to the significant increment of CSCs in xenograft models [14]. Meanwhile, Miao Y et al revealed that TGF-β-responding tumor-initiating stem cells (tSCs) are superior at resisting the transfer of T cells and facilitating tumor relapse using single-cell RNA sequencing (RNA-Seq) and lineage tracing [15]. Thus, it raises our enthusiasm regarding the role of TGF-β in maintaining self-renewal of PCa CSCs.

Speckle-type POZ protein (SPOP) is a bric-a-brac-tram track-broad/poxvirus and zinc finger (BTB/POZ) domain protein that functions as an adaptor for the E3 ubiquitin ligase Cullin3 [16]. SPOP can target various substrates including androgen receptor (AR) [17, 18], steroid receptor coactivator 3 (SRC-3) [19], DEK, TRIM24 [20], ERG [21, 22] and EglN2 [23] for degradation and thus control the proliferation and invasion of PCa. In addition, SPOP inhibits the self-renewal and stem-like characteristics of PCa via the ubiquitin-dependent degradation of NANOG [24] in parallel with the fact that the mutation frequency of *SPOP* gene is up to 15% in PCa with poor prognosis [25]. It has been reported that the expression of SPOP is downregulated in pancreatic cancer, but the underlying mechanism is still unclear [26]. Interestingly, our data by analyzing the TCGA show that expression of *SPOP* gene is downregulated in human PCa tissues, however, little is known about how the transcriptional level of SPOP is tuned in PCa. In this study, we investigated the regulatory mechanism of SPOP expression in PCa especially in terms of CSCs and found that SPOP expression is negatively regulated by SMAD3-mediated TGF-β signaling through the interaction between SMAD3 and its binding elements (SBEs) in the promoter of *SPOP*. Thus, our study reveals a novel role of TGF-β signaling in regulating SPOP expression and resultant PCa stemness.

## RESULTS

### TGF-β signaling is functionally activated in prostate CSCs

**Figure 1:**
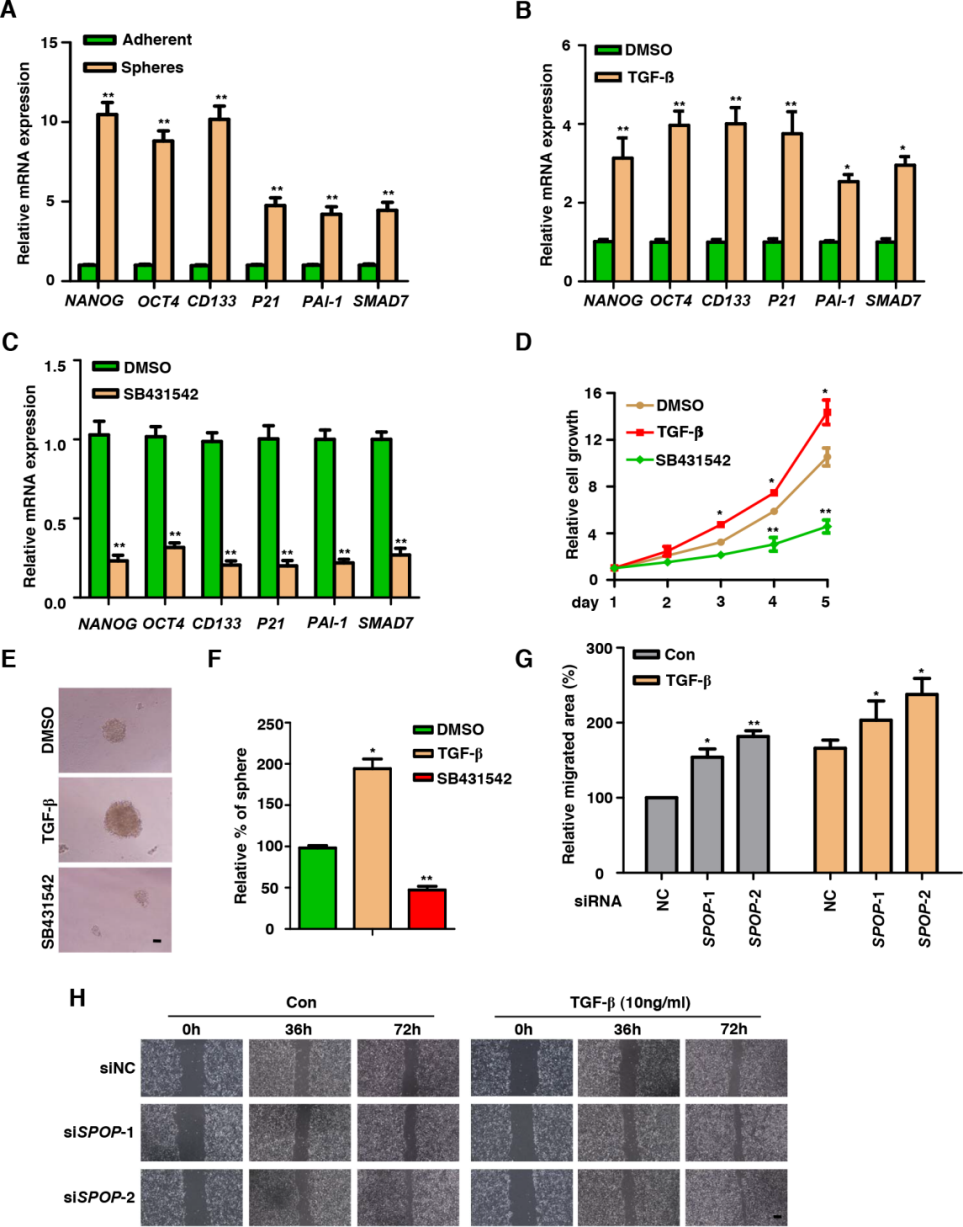
**TGF-β Signaling is functionally active in prostate CSCs.** (**A**) Real-Time PCR analysis of TGF-β Signaling-associated genes in adherent cells versus spheres in DU145 cells. Data are normalized to Actin expression and presented as fold change in gene expression relative to adherent cells. Data are means ± SEM (n=3). **P < 0.01 vs Adherent (Student's *t*-test). (**B**) Analysis the expression of CSCs markers in the treatment of TGF-β (10ng/ml) in DU145 cells via qPCR. Data are normalized to Actin expression and presented as fold change in gene expression relative to the treatment of DMSO. Data are means ± SEM (n=3). *P < 0.05, **P < 0.01 vs DMSO (Student's *t*-test). (**C**) Analysis the expression of CSCs markers in the treatment of SB431542 (10μM) in DU145 cells via qPCR. Data are normalized to Actin expression and presented as fold change in gene expression relative to the treatment of DMSO. Data are means ± SEM (n=3). **P < 0.01 vs DMSO (Student's *t*-test). (**D**) MTT assay of DU145 cells treated with TGF-β (10ng/ml) or SB431542 (10μM) in DU145 cells. Data are means ± SEM (n=3). *P<0.05, **P<0.01 vs DMSO (Student's *t*-test). (**E**) Representative sphere images from each condition of DU145 cells. Scale bar, 100μm. (**F**) Frequency of tumor spheres formed from DU145 cells. Sphere counts are normalized to mock treated spheres. Data are means ± SEM (n=3). *P < 0.05, **P < 0.01 vs DMSO (Student's *t*-test). (**G**, **H**) Wound healing assay of *SPOP* KD PC3 cells. Scale bar, 100μm. Data are means ± SEM (n=3). *P < 0.05, **P < 0.01 vs NC (Student's *t*-test).

TGF-β signaling plays important roles in inducing EMT by enhancing the expression of Snail zinc finger transcription factor family members [27], through which normal or transformed mammary epithelial cells can acquire stem cell-like properties, such as the expression of CD44 and CD133 as well as the capability of forming oncospheres *in vitro* [28]. Thus, it triggers our interest to determine whether TGF-β signaling is upregulated in PCa CSCs by detecting the mRNA expression of its downstream signaling components like *SMAD7*, *PAI-1* and *P21*. The results from real-time PCR demonstrated that TGF-β signaling-related genes are significantly increased in first-passage spheres as compared with cells from which spheres derived (Figure 1A and Supplementary Figure 1A). TGF-β is a cytokine that can radiate signals from a heterodimeric receptor complex formed by the type I (TβRI) and the type II (TβRII) receptors to its downstream signal transducer, SMAD transcription factors, whose activation allows oncogenic instructions to be transmitted by deregulated signals in cancers [29]. Based on our results, we conclude that TGF-β signaling contributes to CSCs turnover in PCa cells via detecting CSCs markers such as *CD133*, *NANOG* and *OCT4* (Figure 1B and Supplementary Figure 1B).

Since SB431542 is a small-molecule inhibitor of ALK5 kinase, a key component in TGF-β signaling axis [30], we explored its role in the induction of CSCs markers of PCa cells such as DU145 and LNCaP. We found that inhibition of TGF-β signaling decreased the expression of CSCs markers (Figure 1C and Supplementary Figure 1C). To test the efficacy of TGF-β on the proliferation of PCa cells, we performed MTT assay upon the treatments of TGF-β and SB431542. Our results showed that TGF-β can promote the proliferation of PCa cells while treatment of TGF-β inhibitor decreased such proliferation in DU145 and LNCaP cells (Figure 1D and Supplementary Figure 1D).

To identify the function of TGF-β on acquiring CSCs properties, we next examined whether TGF-β is capable of enhancing self-renewal capacity of PCa CSCs. In sphere formation assay, the activation of TGF-β signaling resulted in the formation of much larger and more densely populated oncospheres as compared with those of control without TGF-β treatment, which acts in an opposite way that, inhibition of TGF-β impaired the stemness of PCa (Figure 1E and 1F). Furthermore, knockdown of *SPOP* promoted cell migration demonstrated by wound healing assay (Figure 1G, 1H and Supplementary Figure 1E–1H). These results indicate that TGF-β pathway is activated in PCa CSCs and inhibition of TGF-β signaling decreases the proliferation, migration and stemness of PCa.

### *SPOP* expression is regulated by TGF-β signaling in PCa

**Figure 2:**
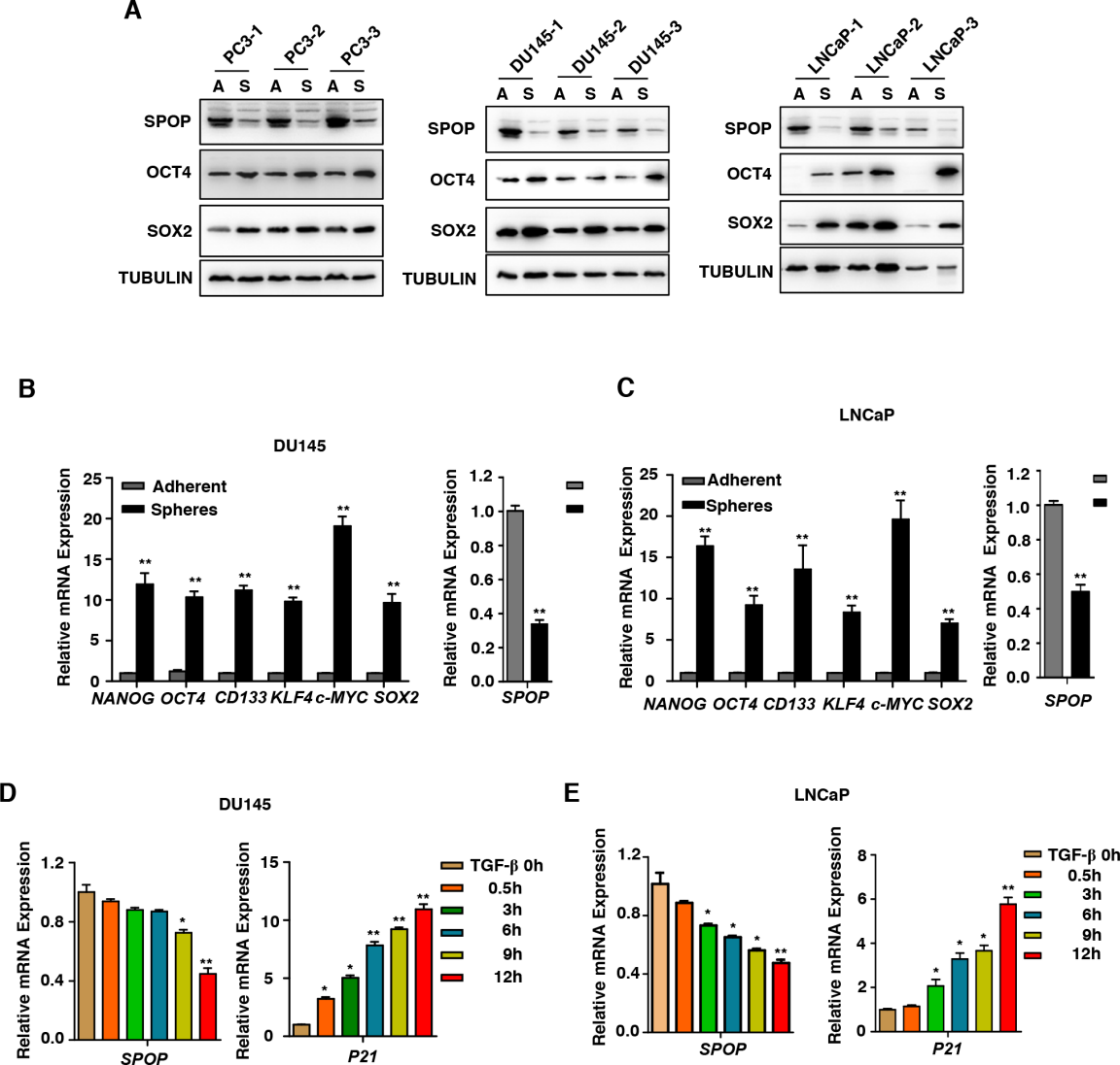
***SPOP* is regulated by TGF-β Signaling in prostate cancer.** (**A**) Western blot analysis the expression of SPOP in the oncospheres in androgen-independent (DU145, PC3) cell lines and androgen-dependent (LNCaP) cell lines. (**B**) Real-Time PCR analysis of *SPOP* and CSCs markers expression in adherent cells versus spheres in DU145 cells. Data are normalized to Actin expression and presented as fold change in gene expression relative to adherent cells. Data are means ± SEM (n=3). **P < 0.01 vs Adherent (Student's *t*-test). (**C**) Real-Time PCR analysis of *SPOP* and CSCs markers expression in adherent cells versus spheres in LNCaP cells. Data are normalized to Actin expression and presented as fold change in gene expression relative to adherent cells. Data are means ± SEM (n=3). **P < 0.01 vs Adherent (Student's *t*-test). (**D**) Analysis the expression of *SPOP* in the treatment of TGF-β (10ng/ml) in DU145 cells. Data are means ± SEM (n=3). *P<0.05, **P < 0.01 vs TGF-β 0h (Student's *t*-test). (**E**) Analysis the expression of *SPOP* in the treatment of TGF-β (10ng/ml) in LNCaP cells. Data are means ± SEM (n=3). *P<0.05, **P < 0.01 vs TGF-β 0h (Student's *t*-test).

Many studies have revealed high-frequency *SPOP* mutation in its MATH domain and these mutations are closely related to the progression of PCa. Interestingly, the TCGA data also show that the expression level of *SPOP* is downregulated in PCa (Figure 4B). Thus, it makes us curious to explore the mechanism underlying such phenomenon. First, we detected the expression of SPOP in PCa oncospheres and found that SPOP is downregulated at both mRNA and protein level in DU145, PC3 and LNCaP cells (Figure 2A–2C).

Next, we examined whether TGF-β takes a part in mediation of *SPOP* expression. We found that under the treatment of TGF-β in PCa cells, the expression level of *SPOP* decreased (Figures 2D and 2E). Based on these data, we conclude that TGF-β plays a key role in diminishing the expression of *SPOP* in PCa oncospheres.

### TGF-β regulates *SPOP* expression via SMAD3

**Figure 3:**
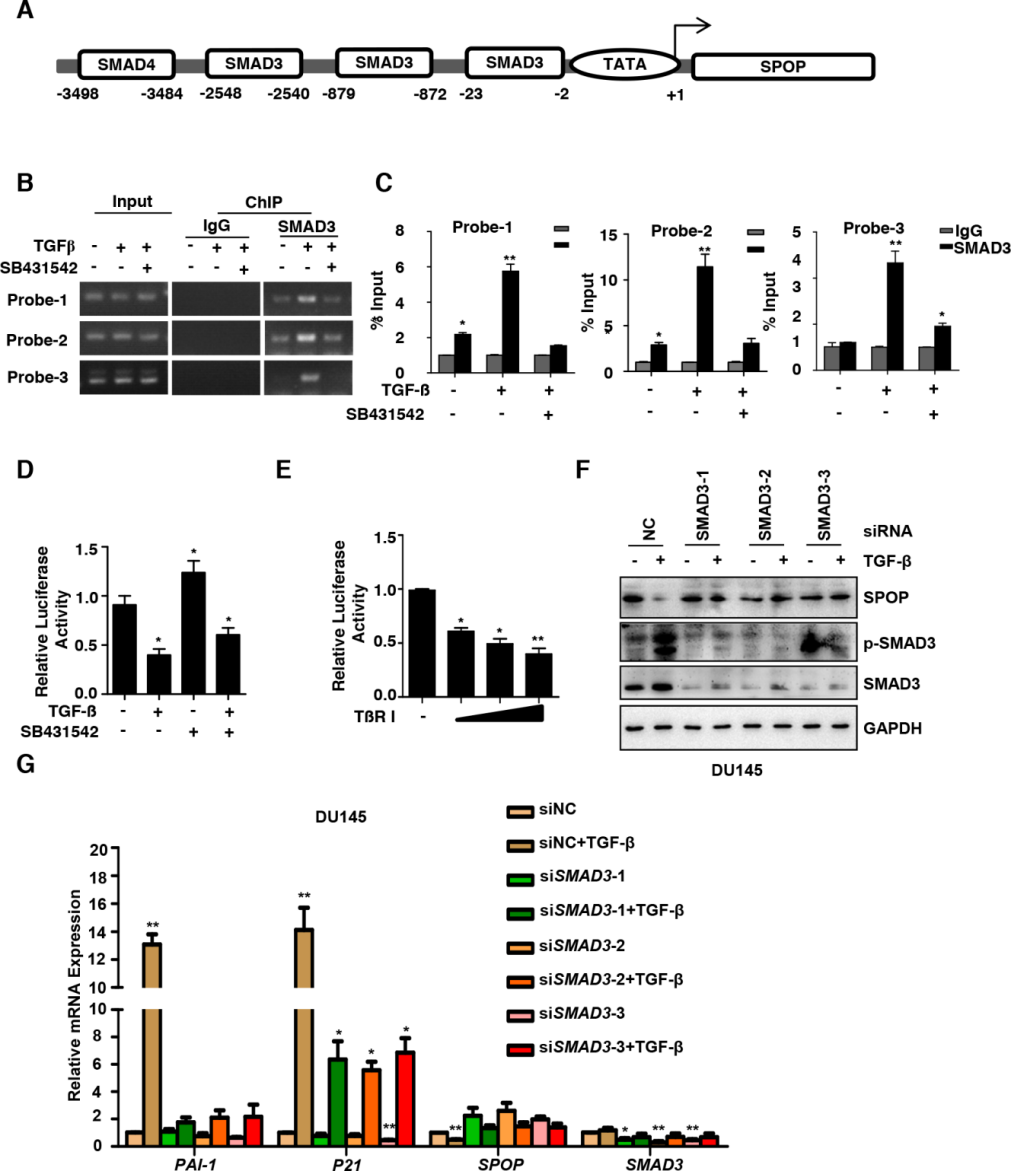
**TGF-β regulates *SPOP* expression through SMAD3.** (**A**) Map of the *SPOP* promoter and the putative SMAD3-binding sites. (**B**) ChIP–PCR analysis of DU145 cells cultured with TGF-β (10ng/ml) or SB431542 (10μM) for 8 hours using anti-SMAD3 antibody and PCR primers. IgG was used as a negative control. (**C**) Enrichment of SMAD3 on the *SPOP* promoter was calculated. Data are means ± SEM (n=3). *P<0.05, **P < 0.01 vs IgG (Student's *t*-test). (**D**) DU145 cells were transfected with *SPOP* gene basic promoter-Luc reporter. After the treatment with TGF-β (10ng/ml) or SB431542 (10μM) for 8 hours, luciferase activity of SPOP were measured. Data are means ± SEM (n=3). *P<0.05 vs TGF-β (-) and SB431542 (-) (Student's *t*-test). (**E**) DU145 cells were transfected with TβRI or vector control, plus the *SPOP* basic promoter-Luc reporter. Luciferase activity of *SPOP* were measured. Data are means ± SEM (n=3). *P<0.05, **P<0.01vs TβRI (-) (Student's *t*-test). (**F**, **G**) Western blot analysis the expression of SPOP upon the knockdown of SMAD3 and the treatment with TGF-β (10ng/ml) for 8 hrs in the DU145 cells (**F**) and the Real-Time PCR analysis of the expression of *SPOP* and TGF-β signaling-associated genes (**G**).

Receptor activated SMADs (R-SMADs, i.e. R-SMAD proteins 2 and 3), are important components of canonical TGF-β signaling pathway, which form complex in the nucleus with DNA-binding co-factors such as SP1 together with transcriptional coactivators or corepressors to regulate gene expression [31]. To understand the molecular underpinnings of how SPOP expression is regulated by TGF-β, we analyzed the potential binding sites of transcriptional factors on *SPOP* promoter using the rVista 2.0 software. We identified three potential SMAD3-binding sites in *SPOP* promoter (Figure 3A). Next, we examined whether the expression level of *SPOP* is regulated by SMAD3. Chromatin immunoprecipitation (ChIP) analysis was performed and revealed that SMAD3 was preferentially enriched at the *SPOP* promoter under the treatment of TGF-β, which was significantly reduced when PCa cells were treated with SB431542 (Figure 3B and 3C). In addition, TGF-β treatment leads to a downregulation of SPOP at the transcriptional level using promoter-driven luciferase reporter gene assay. Our data demonstrated that inhibition of TGF-β signaling increased the mRNA level of *SPOP* (Figure 3D). Moreover, ectopic expression of TβRI remarkably decreased the mRNA level of *SPOP* in a dose-dependent manner (Figure 3E) while treatment of TGF-β has no effect on the mRNA expression of *SPOP* when SMAD3 is knocked down (Figure 3F and 3G, Supplementary Figure 3A and 3B).

### *SPOP* expression is downregulated in PCa.

**Figure 4:**
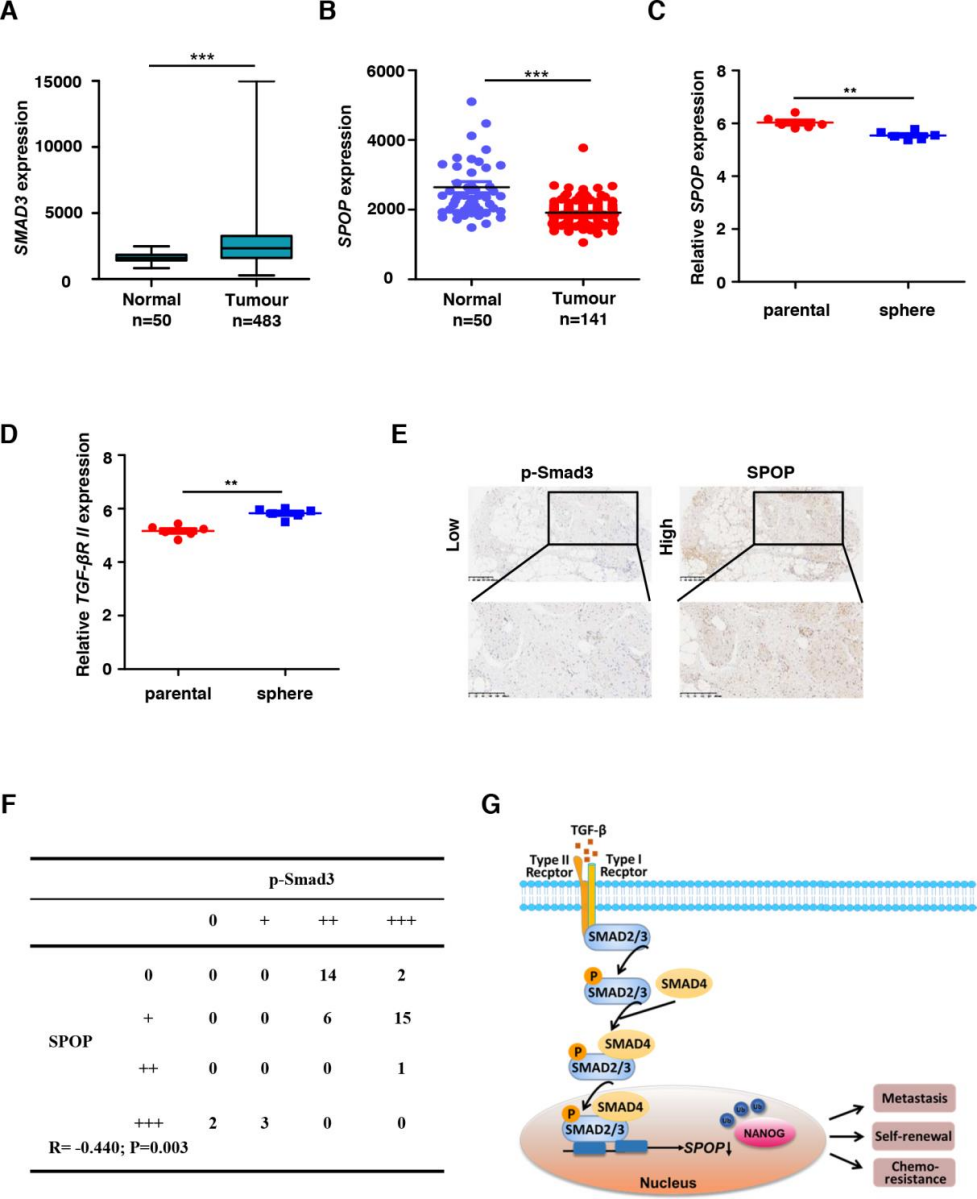
***SPOP* expression is downregulated in human proatate cancer.** (**A**) *SMAD3* expression levels in normal tissue and primary tumor of prostate through the TCGA data. Expression levels are presented as boxplots and were compared using an unpaired Student's *t*-test, ***P<0.001. (**B**) *SPOP* expression levels in normal tissue and primary tumor of prostate through the TCGA data. Expression levels are presented as boxplots and were compared using an unpaired Student's *t*-test, ***P<0.001. (**C**) Relative *SPOP* expression levels in parental sample and sphere sample of prostate cancer through the GEO data GSE19713. Expression levels are presented as boxplots and were compared using an unpaired Student's *t*-test, **P<0.01. (**D**) Relative TGFβR II expression levels in parental sample and sphere sample of prostate cancer through the GEO data GSE19713. Expression levels are presented as boxplots and were compared using an unpaired Student's *t*-test, **P<0.01. (**E**) Human prostate tumor specimens were stained with p-SMAD3 and SPOP separately using an IHC staining assay. Representative examples are shown. (**F**) The correlation between SPOP and p-SMAD3 protein levels in the human prostate tumor tissue array is shown. Statistical significance was determined by a χ2 test. R indicates the correlation coefficient. (**G**) Model for TGF-β signaling negatively regulates *SPOP* expression.

To investigate the clinic-pathological and prognostic value of SPOP expression in PCa, we detected the difference of SPOP expression between normal tissues and primary tumors. We also analyzed the expression of SMAD3, SPOP and TGFβR II using clinic PCa samples from TCGA data. We noticed an increased expression level of SMAD3 concomitant with a SPOP reduction in those samples (Figure 4A and 4B), which is consistent with our results (Figure 3F, 3G and Supplementary Figure 3A). In accordance with that, we also observed a reduction in *SPOP* expression together with an upregulation of *TGFβR II* in PCa oncospheres (Figure 4C and 4D).

Next, we examined whether SPOP expression is correlated to the expression levels of p-SMAD3 in PCa using IHC. Our data indicated that SPOP positively correlated with the expressions of p-SMAD3 (Figure 4E), which is statistically significant when their IHC staining was quantified (Figure 4F), highlighting the clinical importance of SPOP expression in PCa.

## DISCUSSION

TGF-β has been shown to enrich CD44^high^ populated CSCs via EMT [28, 32]. Recent studies demonstrate that TGF-β regulated genes are tightly associated with PCa bone metastases in parallel with the fact that inhibition of TGF-β signaling minimizes the development of bone metastases [11–13]. Accumulating evidence also indicate an intimate relationship between CSCs and PCa initiation, progression and resistance to chemotherapy [1, 2]. In the present study, we demonstrate that the TGF-β signaling plays a critical tumor-promoting role in PCa. Inhibition of TGF-β signaling reduced the formation of CSC-like oncospheres derived from PCa cells, suggesting a potential role of TGF-β signaling axis in the induction of PCa oncospheres (Figure 1E and 1F). Our data hint an alternative approach of TGF-β signaling that critically influence the acquirement of stem properties in both PCa cells and clinic PCa tissues.

It has been reported that E3 ubiquitin ligase SPOP acts as a tumor suppressor in PCa [24, 33]. Our data indicate that the expression level of *SPOP* is much lower in PCa. Interestingly, we also found that *SPOP* is downregulated in PCa CSCs, which is manipulated by TGF-β signaling (Figure 2A–2C). In response to TGF-β-mediated dimerization between TGF-β receptor I (TGF-βRI) and TGF-βRII, receptor-regulated SMADs (SMAD2/3) are phosphorylated, interact with SMAD4, and translocate to the nucleus where they form a complex controlling gene transcription with some DNA-binding partners and transcriptional co-activators or co-repressors [34]. To our surprise, despite the fact that AR has a cross-talk role with TGF-β signaling and plays an important role in the development and progression of PCa and its androgen-independent transformation [35], we confirmed that TGF-β regulates the gene expression of SPOP via SMAD3 in both androgen-independent (DU145, PC3) cell lines and androgen-dependent (LNCaP) cell line (Figures 2D, 2E, Supplementary Figure 2A and 2B). The same effect regardless of androgen-independence and -dependence may be due to the direct interaction between *SMAD3* and *SPOP* promoter, which is separated from AR signaling. However, which transcriptional components that SMAD3 recruits during such process need to be addressed in the future.

Taken together, our study identifies a novel mechanism of TGF-β signaling in tumorigenesis through downregulating the expression of SPOP, a potential tumor suppressor, which may lead to the upregulation of NANOG as we previously reported [24] and consequently enhanced stemness in PCa. We summarized the essence of our findings as a novel model described in Figure 4G. These results suggest that the newly-identified TGF-β / SPOP signaling node may serve as potential therapeutic target for the treatment of cancers by eliminating the stemness of PCa.

## MATERIALS AND METHODS

### Plasmids and antibodies

Constructs were generated by standard molecular cloning method. *SPOP* basic promoter luciferase reporter was cloned into the PGL3 basic vector. *TβR I* and *Renilla* were cloned into pCDNA3.1 vector. All the vectors were confirmed using DNA sequencing. The anti-SOX2 (sc-20088, 1:3000) antibody was obtained from Epitomics. The anti-NANOG (AB5731, 1:500) antibody was purchased from Millipore. The anti-Tubullin (2148S, 1:1000), anti-GAPDH (5174S), anti-p-SMAD3 (9520S), anti-SMAD3 (9523S) antibodies were obtained from Cell Signaling Technology. The anti-OCT4 (ab181557) antibody was obtained from Abcam. TGF-β (10ng/ml) was purchased from Sigma, SB431542 (10 μM) was purchase from Selleck.

### Cell culture and transfection

Human embryonic kidney 293T cells (HEK293T cells, American Type Culture Collection) was cultured in Dulbecco's modified Eagle's medium (DMEM) (Gibco). DU145 cells (American Type Culture Collection), PC3 cells (American Type Culture Collection) and LNCaP cells (American Type Culture Collection) were cultured in 1640 medium (Gibco), supplemented with 10% heat-inactivated fetal bovine serum (FBS) at 37 °C in 5% CO2. The identity of all the cell lines has been authenticated by the American Type Culture Collection through the STR profiling.

Transfections were performed using calcium phosphate-DNA coprecipitation for 293T cells and SunbioTrans-EZ for DU145 cells and LNCaP cells (Shanghai Sunbio Medical Biotechnology Co., Ltd.). DU145 cells and LNCaP cells were transfected with siRNA oligonucleotides using Lipofectamine2000.

### Luciferase reporter assay

HEK293T cells were transiently co-transfected with firefly luciferase reporter vectors, effector vectors and the renilla luciferase vector. After 36 h, cells were collected in lysis buffer (25mM Tris-Cl (pH 7.8), 25mM dithiothreitol (DTT), 2mM 1,2-diaminocyclo-hoxaneN,N,N,N’-tetracetic acid, 10% glycerol and 1% Triton X-100), and luciferase assays were performed using the dual-luciferase reporter assay system (Promega).

### Real-time qPCR

Total RNA was Trizol-extracted, column-purified and reverse-transcribed using PrimeScript 1^st^ Strand Cdna Synthesis kit (Takara). All qPCR analyses were performed using Fast SYBR Green (Takara).

### Proliferation assay

For MTT assay, DU145cells and LNCaP cells were seeded in a 96-well plate. Cells were harvested every 24hours, the MTT solution was added for 4 hours. The reactions were stopped by addition of dimethyl sulfoxide (DMSO) solution for 20 minutes, and the samples were measured at 490 nm. Three independent experiments were carried out.

### Sphere formation assay

Oncospheres were enriched from DU145 cells. Single-cell suspension of DU145 cells (200 cells per well) were plated on 96-well ultra-low Attachment Plates (Corning Incorporated, catalog number: 3474) and cultured in Dulbecco’s Modified Eagle’s Medium/F12 (Gibco) supplemented with 5μg/ml insulin (Sigma), 20ng/ml EGF (Sigma), 1:50 B27 (Gibco), 10ng/ml bFGF, and 0.4% BSA for 10 days alone. Floating spheres that grew in 2 weeks were counted. Tumor spheres were visualized under phase contrast microscope, photographed and counted and represented graphically. Spheres were digested with trypsin 0.05% EDTA and filtered through a 40-mm filter.

### TCGA data analysis

Level 3 data for mRNA expression from TCGA were downloaded and processed using standard methods. mRNA expression was measured using the Illumina HiSeq 2000 RNA Sequencing version 2 program. Gene expression was analyzed using two-class unpaired significance analysis of microarrays (SAM) (http://statweb.stanford.edu/~tibs/SAM/) for the indicated tumors versus normal samples. Differences in expression were considered to be statistically significant when the fold change>2 and q<0.05. Kaplan-Meier plot was analyzed from the PrognoScan database (http://www.abren.net/PrognoScan/).

### Statistical analysis

Statistical analyses were performed with a two-tailed unpaired Student’s t-test. The data are presented as the means ± SEM. The mean was calculated from truly independent experiments. P-values < 0.05 were considered statistically significant.

### IHC staining of human prostate cancer tissues

The human prostate cancer tumor tissue arrays were provided by Changhai hospital (Shanghai, China). The arrays were stained by IHC with SPOP, p-Smad3 and NANOG-specific antibodies using the Histostain-plus IHC Kit (Miao Tong Biological Science and Technology Co., LTD, Shanghai, China). The stained slides were examined under a microscope, and images were acquired.

## Supplementary Material

Supplementary Figures
