# Supplementary material for: TGF-β signaling regulates SPOP expression and promotes prostate cancer cell stemness
Source: Aging (Albany NY). 2020 May 1;12(9):7747–60. doi: 10.18632/aging.103085 (PMC7244043; doi:10.18632/aging.103085)
Supplement: Supplementary Figures [file aging-12-103085-s001..pdf]

SUPPLEMENTARY FIGURES

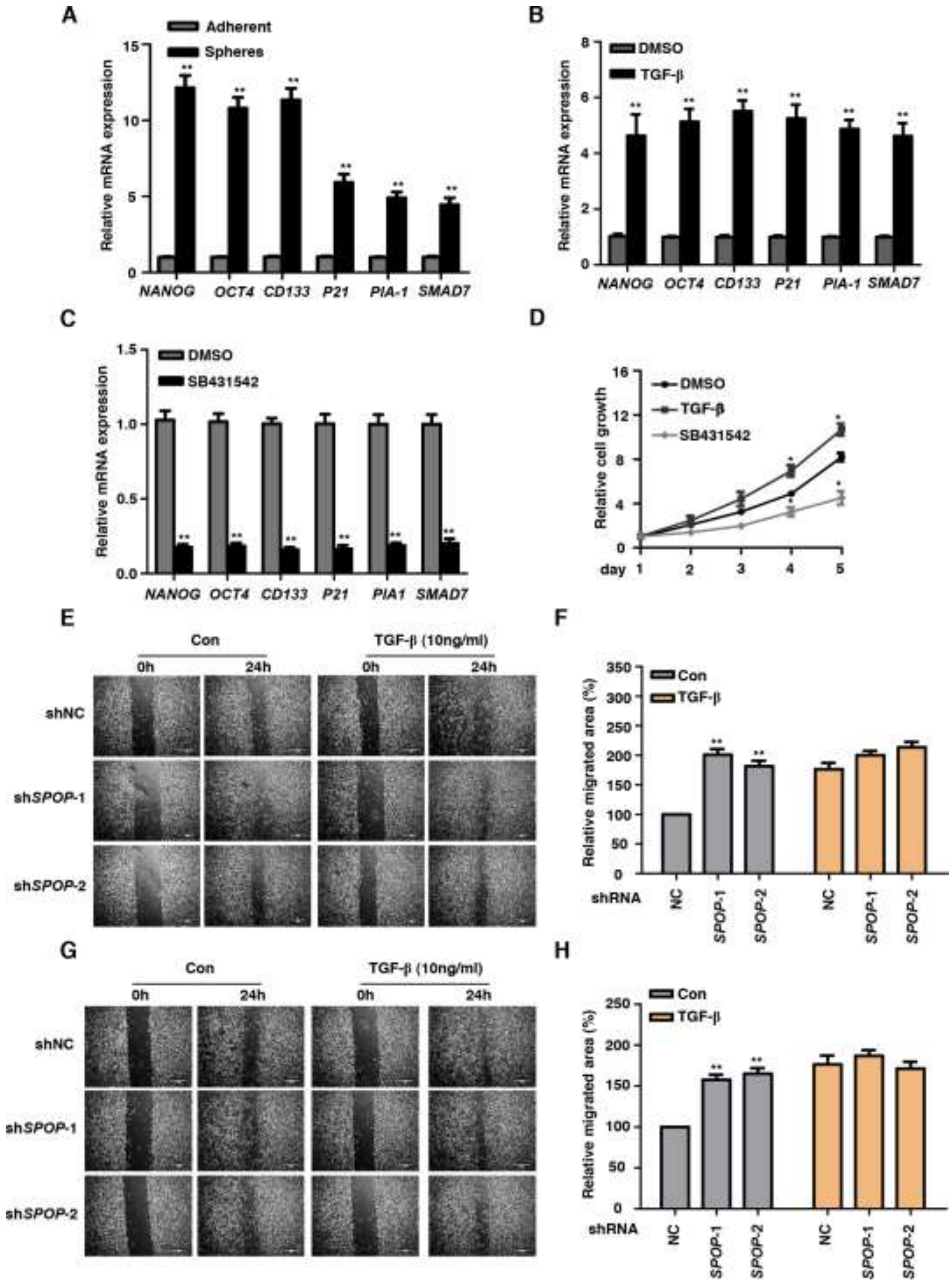

Supplementary Figure 1. TGF-β Signaling is functionally active in prostate CSCs. (**A**) Real-Time PCR analysis of TGF-β Signaling-associated genes in adherent cells versus spheres in LNCaP cells. Data are normalized to Actin expression and presented as fold change in gene expression relative to adherent cells. Data are means ± SEM (n=3). $**P < 0.01$ vs Adherent (Student's *t*-test). (**B**) Analysis the expression of CSCs markers in the treatment of TGF-β (10ng/ml) in LNCaP cells via qPCR. Data are normalized to Actin expression and presented as fold change in gene expression relative to the treatment of DMSO. Data are means ± SEM (n=3). $**P < 0.01$ vs DMSO (Student's *t*-test). (**C**) Analysis the expression of CSCs markers in the treatment of SB431542 (10μM) in LNCaP cells via qPCR. Data are normalized to Actin expression and presented as fold change in gene expression relative to the treatment of DMSO. Data are means ± SEM (n=3). $**P < 0.01$ vs

DMSO (Student's *t*-test). (**D**) MTT assay of LNCaP cells treated with TGF-β (10ng/ml) or SB431542 (10μM) in LNCaP cells. Data are means ± SEM (n=3). **P*<0.05, ***P*<0.01 vs DMSO (Student's *t*-test). (**E**, **F**) Wound healing assay of SPOP KD DU145 cells. Scale bar, 100μm. Data are means ± SEM (n=3). ***P* < 0.01 vs NC (Student's *t*-test). (**G**, **H**) Wound healing assay of SPOP KD LNCaP cells. Scale bar, 100μm. Data are means ± SEM (n=3). ***P* < 0.01 vs NC (Student's *t*-test).

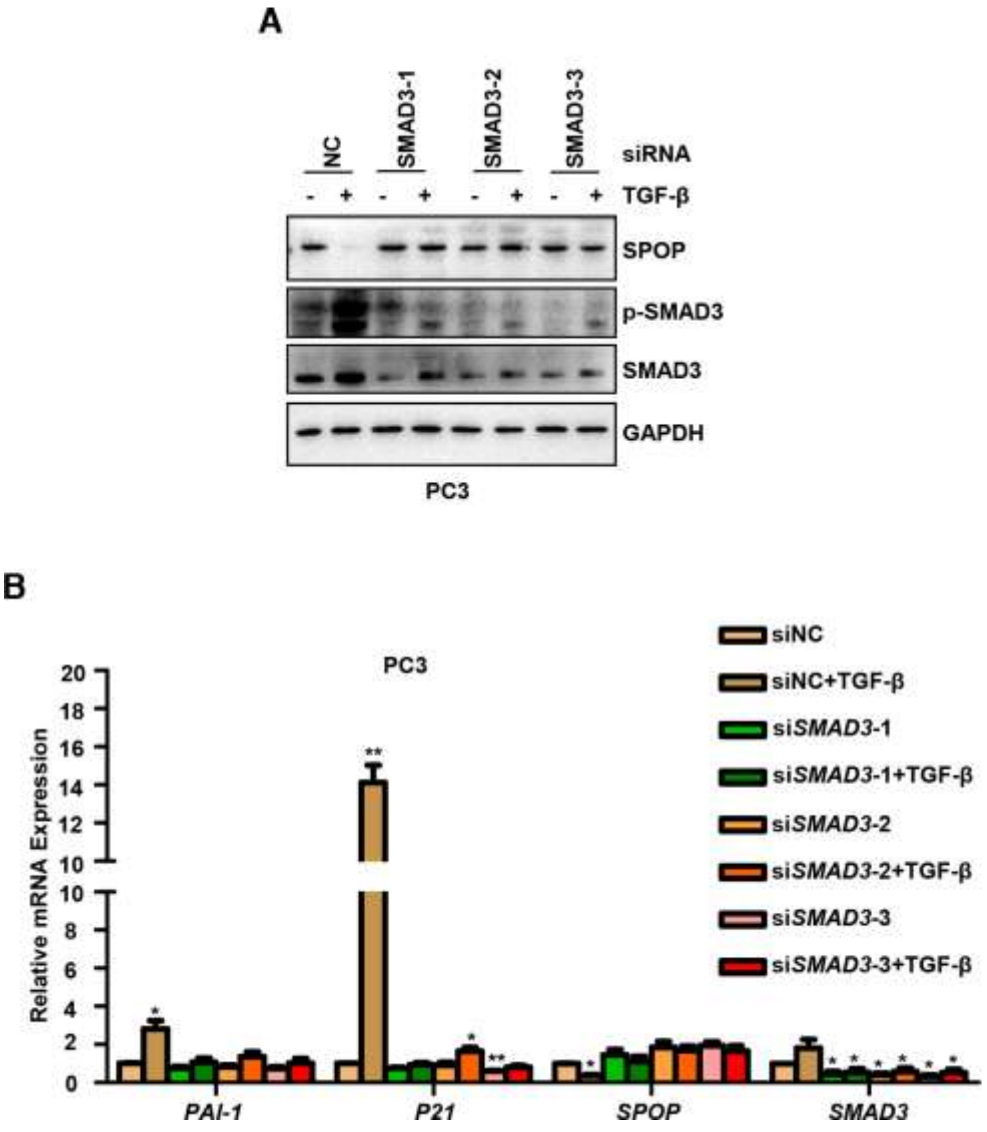

Supplementary Figure 2. TGF-β regulates SPOP expression through SMAD3. (**A**) Western blot analysis the expression of SPOP upon the knockdown of SMAD3 and the treatment with TGF-β (10ng/ml) for 8 hrs in the PC3 cells. (**B**) Real-Time PCR analysis of the expression of *SPOP* and TGF-β Signaling-associated genes.

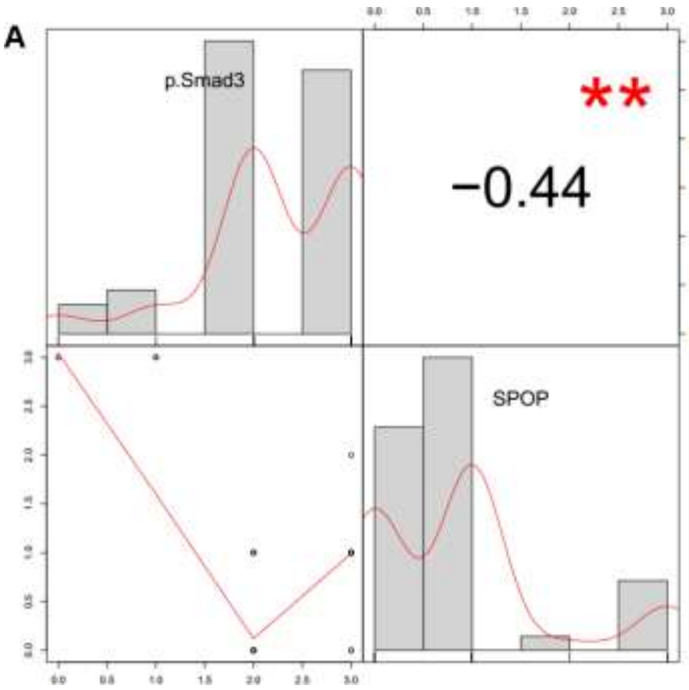

Supplementary Figure 3. The negative correlation between TGF-β signaling and SPOP expression in human prostate tumor specimens. (**A**) The corPlot of correlation between TGF-β signaling and SPOP expression in human prostate tumor specimens.
